# Probing protein–lipid interactions by hydrogen/deuterium exchange-mass spectrometry: advances, challenges and future directions

**DOI:** 10.1039/d6sc04457c

**Published:** 2026-08-07

**Authors:** Artemis Lioupi, Agni F. M. Gavriilidou, Argyris Politis

**Affiliations:** a Institute for Bioinnovation, Biomedical Sciences Research Center “Alexander Fleming” Vari 16672 Greece argyris.politis@manchester.ac.uk politis@fleming.gr; b Faculty of Biology, Medicine and Health, School of Biological Sciences, The University of Manchester Manchester M13 9PT UK; c Manchester Institute of Biotechnology, University of Manchester Princess Street Manchester M1 7DN UK

## Abstract

Membrane proteins constitute over 30% of the human proteome and represent more than 60% of drug targets, making them of critical interest in pharmaceutical discovery. Membrane proteins function within complex lipid environments that actively regulate their structure, dynamics, and activity. Hydrogen–deuterium exchange mass spectrometry (HDX-MS) has emerged as a powerful approach for probing membrane protein dynamics in solution and native-like assemblies, including nanodiscs, SMALPs, and liposomes, paving the way to study integral membrane proteins within the context of living cells. In this perspective, we discuss HDX-MS analysis of membrane protein–lipid interactions, particularly key challenges such as lipid-induced ion suppression and chromatographic interference, and highlight recent advances in lipid removal strategies, subzero chromatography, mass spectrometry/ion mobility, and the integration of molecular dynamics simulations and artificial intelligence. We further emphasise the synergy of HDX-MS with lipidomics and native MS to better understand the interplay between lipid composition, binding stoichiometry, and structural dynamics. Together, these approaches establish an emerging multidimensional framework for understanding membrane protein–lipid interplay under physiologically relevant conditions.

## Abstract 2

Hydrogen–deuterium exchange mass spectrometry has emerged as a powerful approach for probing membrane protein dynamics in solution and native-like assemblies, paving the way to study integral membrane proteins within the context of living cells.

## Graphical abstract

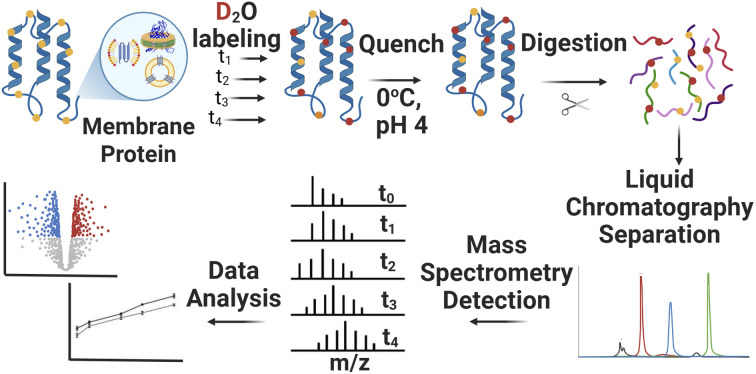

## 1 Introduction

Membrane protein function and associated mechanisms are profoundly influenced by the surrounding lipid environment, which is highly dynamic, and any perturbation of membrane lipids can significantly affect the structure and function of membrane proteins. Capturing membrane protein–lipid interactions without perturbing the native lipid environment is an ongoing challenge. Traditional structural biology methods often fail to capture the dynamic interplay between membrane proteins and lipids due to challenges in solubilisation, conformational heterogeneity, and sample complexity. As such, characterising membrane proteins as close to their native-like bilayer environments as possible will help uncover many new roles for lipids. Each approach has distinct limitations; for instance, the natural lipid environment is frequently removed for X-ray crystallography studies,^1^ and cryo-electron microscopy (cryo-EM) struggles with small protein complexes^2^ while nuclear magnetic resonance (NMR) is limited to smaller proteins and requires extensive sample preparation.^3^ Moreover, some structural studies require the flexible termini and dynamic loops to be removed, or the membrane protein to be stabilised with fusion proteins and mutagenesis to increase the overall stability of the native fold, perturbing the natural environment.^4^ To overcome these challenges and gain a more complete picture of how membrane proteins and lipids interact, researchers often use a combination of analytical techniques.

Over the last three decades, mass spectrometry (MS) has emerged as a key approach for structural biology. MS can be used to directly probe the structure and dynamics of protein assemblies.^5^ Native MS has emerged as a complementary analytical approach in studying membrane proteins due to its compatibility with a wide mass range, with molecular weights up to MDa, high speed, sensitivity and its label-free analysis.^6^ Membrane proteins exist in distinct molecular forms that can vary in covalent modifications, stoichiometry, and conformation, and native MS can help inform on this heterogeneity because it avoids ensemble averaging by separating distinct molecular populations prior to detection, rather than reporting only a single ensemble-averaged signal. Liquid chromatography (LC)-MS lipidomics can analyse and determine the lipid structure by integrating separation and fragmentation. Slight differences in lipid structure are important for protein conformational state and activity. Thus, identifying the lipid structure is crucial for explaining protein function and understanding human health and disease.^7^

Hydrogen/deuterium mass spectrometry (HDX-MS) is a solution-phase method to monitor conformational dynamics and solvent accessibility at peptide-level resolution, and more recently, single amino acid level resolution has been demonstrated.^8^ It can be used to investigate protein conformational changes upon substrate binding, ligand interactions, and mutations among others. HDX-MS is commonly used for probing the structural dynamics of soluble proteins, where experimental workflows are more established. Extending HDX-MS to the study of lipid interactions with membrane proteins is also possible and can provide direct insights into lipid-binding sites as well as lipid-induced stabilisation, modulation, or allosteric regulation. However, such experiments are considerably more technically demanding and are not yet routine or fully standardised, as they require careful control of membrane mimetics and sample handling to preserve native-like interactions.

**Fig. 1:**
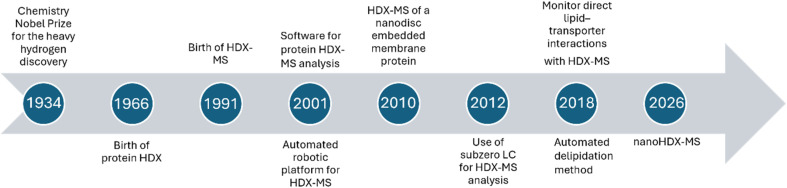
Timeline showing key milestones on HDX-MS. Birth of protein HDX by Hvidt A. *et al.*,^15^ birth of protein HDX-MS^16^ by coupling HDX with MS resolving power for protein structure analysis, emergence of an automated robotic platform^17^ by integrating sample handling and LC-MS analysis, first fully integrated software for HDX-MS data analysis^17,18^ for extraction of deuterium incorporation from isotopic distributions, first study of a nanodisc-embedded transporter,^13^ implementation of subzero LC for HDX-MS analysis^19^ to minimize back-exchange and allow long LC separations, monitor lipid-transporter interactions from a nanodisc,^12^ first automated delipidation method^20^ using zirconium oxide beads and filtration, implementation of nanoHDX-MS^21^ to significantly reduce sample consumption.

Most studies are performed in detergent micelles, which enable solubilisation but do not fully reproduce the native lipid bilayer environment and, in some cases, alter conformational equilibria.^9–11^ As a result, increasing effort has been directed toward studying membrane proteins in progressively more native-like systems, including liposomes and nanodiscs.^12,13^ While these approaches better preserve protein–lipid interactions, they introduce additional experimental complexity related to lipid interference, sample heterogeneity, and back-exchange control. The major milestones and methodological advances in applying HDX-MS to membrane protein structural dynamics are summarised in Fig. 1.

In this perspective, we describe recent technical developments, discuss how HDX-MS can be leveraged to investigate membrane protein–lipid interactions, outline the remaining challenges, and highlight how complementary MS techniques can further enhance our understanding of membrane protein–lipid interactions. The focus is on how these integrated approaches are helping to deepen insight into the complex nature of membrane protein–lipid assemblies. We provide an overview of the potential of these emerging MS-based methods by assessing their likely role in conjunction with other structural biology approaches commonly applied to membrane proteins and their complexes. We will conclude by offering a future perspective discussing how continued improvements in sample preparation, separation technologies, MS sensitivity and data analysis will enable HDX-MS to address increasingly sophisticated and exciting biological questions. Notably, *in vivo* HDX-MS has already been demonstrated,^14^ although routine implementation and broad applications remain challenging. We therefore discuss how emerging developments may overcome these limitations and enable routine characterisation of protein dynamics and interactions directly within living cells. Achieving the next level that includes integration of other MS, and not only, will also be equally challenging and may be associated with further technical limitations and lack of expertise. Synergetic MS data can be brought together to build detailed structural models of membrane protein complexes, giving a clearer picture of how they function in their native environment.

## 2 Membrane protein–lipid interactions: a functional overview

### 2.1 Biological relevance: lipid regulation, structural stability, signalling

Of the many factors that govern the stability and function of membrane proteins, the local lipid environment is perhaps the most critical. Effects of lipid structure on membrane protein function can be described in molecular terms, with hydrophobic effects, hydrogen bonding, and charge interactions, or in physical terms, with properties such as lipid fluidity, membrane curvature and tension.

Lipids fulfil three principal functions.^22^ First, they serve as energy storage molecules. Second, polar lipids form the structural matrix of cellular membranes. These molecules contain both hydrophobic and hydrophilic regions; the tendency of the hydrophobic moieties to self-associate and of the hydrophilic moieties to interact with aqueous environments and with each other constitutes the physical basis for the spontaneous formation of membrane bilayers. Third, specific lipids are essential for protein function, where they contribute to structural stability, regulate activity, and participate in signalling processes.^23^

Based on the residence time and the degree of affinity for membrane proteins, lipids interacting with membrane proteins can be classified into three categories: bulk lipids, annular lipids, and non-annular lipids.^24^ The lipid molecules in a membrane that are not in contact with a protein are usually referred to as bulk lipids. Most lipids in contact with a membrane protein act as a solvent for the protein, interacting with it in a largely nonspecific manner.^25^ The solvent lipids have been referred to as annular lipids, to denote the fact that they form an annular shell of lipid around the protein.^26^ Annular lipids are therefore often lost during solubilization and/or purification of membrane proteins. The rapid exchange of lipid molecules between the annular shell and the bulk phase suggests that lipid–protein interactions are non-specific and dynamic in nature.^27^ Distinguishing lipids that induce a direct effect on membrane proteins from loosely associated ones remains a challenging task and raises questions about whether or not the lipid annulus is constant in composition or variable. A small number of lipid molecules, referred to as non-annular lipids, interact with much greater specificity with the protein; these lipid molecules are often essential for the activity of the protein.^24^ Owing to their affinity, non-annular lipids often remain tightly bound to membrane proteins, even following purification and crystallisation in detergent micelles. The effect of non-annular lipids on membrane protein structure and function depends on the degree of hydrophobic matching between the membrane core and the surrounding lipid acyl chain length and depends on the distribution of charges at the protein–lipid interface. Diverse activities have been proposed for these non-annular lipids in modulating the structure and function of membrane proteins. For instance, lipids can act as cofactors,^28^ as allosteric regulators of membrane protein activity,^29^ or as regulators of enzymatic functions of membrane proteins.^30^

All membrane lipids are amphipathic in nature, consisting of both hydrophilic (phosphate and other headgroups) and hydrophobic (acyl chains) moieties. Lipids found in protein membranes fall into four main classes—glycerophospholipids, sphingolipids, glycolipids, and sterols, and their concentration depends largely upon the cell type and organelle.^31^ In general, the most abundant class is the glycerophospholipids, also known simply as phospholipids (PLs), while sphingolipids constitute the second major lipid class.^32^ Glycolipids are sugar-containing lipids, located at the extracellular side of the membrane, serving as both markers for cellular recognition and signals for cells to grow or divide. The sterol category is subdivided primarily on the basis of biological function, with cholesterol being one of the most predominant sterols. Cholesterol is present in higher eukaryotes but is absent from prokaryotes and is able to modify the fluidity of a membrane in a concentration-dependent manner.^33^ Cholesterol has been shown to modulate conformation, stability, and signalling activity of G protein-coupled receptors (GPCRs),^34,35^ one of the largest protein families in mammals. Some lipids have charged headgroups (PLs and sphingolipids (SP)), some contain reactive amines (phosphatidylethanolamine (PE) and phosphatidylserine (PS)), whereas a few others are relatively bulky (cardiolipin (CDL), phosphatidylinositol (PI), phosphatidylcholine (PC), and phosphoinositol 4,5-bisphosphate (PIP2)). Therefore, orientation of lipids, charge density, and movement of headgroups are important for lipid–protein binding and membrane topology.^36^

### 2.2 Role of native-like environments

Proteins and lipids are not separate entities and should ideally be studied together. However, studying membrane proteins with MS *in situ* or *in vivo* is a challenging task and is in its very early stages^14,37,38^ due to their low abundance, instability, heterogeneous and dynamic character. The majority of membrane protein studies have been performed in detergents/micelles due to their ease in use. However, lately several possibilities that mimic natural membranes, such as nanodiscs, bicelles, liposomes and styrene-maleic acid copolymers (SMALPs) have been developed.

Micelle-forming detergents provide an amphipathic environment that can mimic lipid bilayers and are currently the most widely used membrane mimetics. Detergent monomers in solution self-assemble at and above the critical micelle concentration to form micelles. Micelles adopt globular shapes (*e.g.*, spheres, ellipsoids, and cylinders) of various sizes, determined by the detergent head group structure and alkyl chain length.^39^ However, the formation of a soluble protein-detergent complex currently relies on empirical screening of detergents until the right combination of protein and detergent is found for a stable and functional protein.^40^ In many cases, it is preferable to incorporate the proteins into a more native-like membrane environment, as notable structural and dynamic differences have been observed between detergent-solubilised and membrane-embedded forms.^9–11^

Nanodiscs provide a disc-shaped environment consisting of PLs and surrounded by a membrane scaffold protein (MSP). Nanodiscs have become a prominent membrane mimetic system for studying membrane proteins, they are highly tunable, allow precise control over size and lipid composition with nanoscale bilayer structure that mimics the general structure of biological membranes.^41^ A range of MSP constructs is available to generate nanodiscs of variable diameters, accommodating membrane proteins of various sizes.^42^ In the context of HDX-MS, nanodiscs provide an invaluable tool for the study of membrane proteins in a native-like lipid environment,^43^ the protein being accessible for ligands/substrates from both sides. However, the contaminating peptides from MSP increase MS spectral complexity, requiring additional sample clean-up steps that often complicate HDX-MS workflows. Saposin A (Sap A) nanodiscs are a lipoprotein nanoparticle system that uses Sap A peptides to surround the membrane protein in a lipid bilayer.^44^ The major advantage of Sap A nanodiscs is their adaptability to any protein size, in contrast with MSP nanodiscs, where optimisation of the different-sized MSP constructs is required.^45^

Bicelles are formed from mixed PLs that can assemble into bilayer-like structures. They mainly comprise long-chain PLs that constitute the bilayer-like region and short-chain PLs or detergents that constitute the rim region to close the disc structure.^46^ The ease of reconstitution and the absence of a lipid-removal clean-up step further enhance their suitability for HDX-MS studies. On the other hand, the presence of detergent may perturb the membrane protein structure and function.

SMALPs are mixtures of SMA copolymers and lipids that readily incorporate into cellular membranes or vesicles and rapidly solubilise them into nanodisc structures from biomembranes.^47^ This approach is completely detergent-free. However, their sensitivity to low pH and divalent cations restricts their true potential in HDX-MS applications.^48^ Recently, the emergence of SMA-like copolymers, shown to be stable under these conditions, could potentially overcome these challenges.^49^

Liposomes are PL bilayer vesicles that resemble natural PL membranes. Due to their variability in size, composition, and amphiphilic character, they can be applied to most membrane proteins.^50^ Liposomes are very close to native membranes in curvature, offer tunable lipid composition, and can accommodate multicomponent systems owing to their large size. Nevertheless, the heterogeneity in orientation of the reconstituted protein may complicate the interpretation and reproducibility of the results. One advantage of using liposomes for HDX-MS of membrane proteins,^51,52^ is the absence of a fixed lipid-to-protein ratio, allowing proteins to undergo conformational changes and variations in size more freely, closely mimicking their behavior in a native membrane environment.

*In situ* characterisation of membrane proteins avoids reconstitution systems that may perturb membrane protein structure and function, thereby preserving the native lipid environment. A few studies have demonstrated *in situ* HDX-MS of integral membrane proteins,^14,53–56^ showing how protein dynamics are in their natural environment. *In situ* studies, however, are still labour-intensive, including intensive sample cleanup, protein precipitation, and introducing opportunities for measurement error between replicates.

From the aforementioned native-like environments, nanodiscs are considered particularly well-suited for HDX-MS. Apart from providing a native phospholipid bilayer environment, they remain homogeneous, stable and relatively small in size, making their clean-up easier and more standardised for HDX-MS workflows compared to a mimetic where the lipid composition cannot be controlled. Nanodiscs also allow studying membrane proteins in different lipid compositions, which allows following protein dynamics in different environments.

## 3 Challenges of HDX-MS for membrane proteins

### 3.1 Lipid filtration

**Fig. 2:**
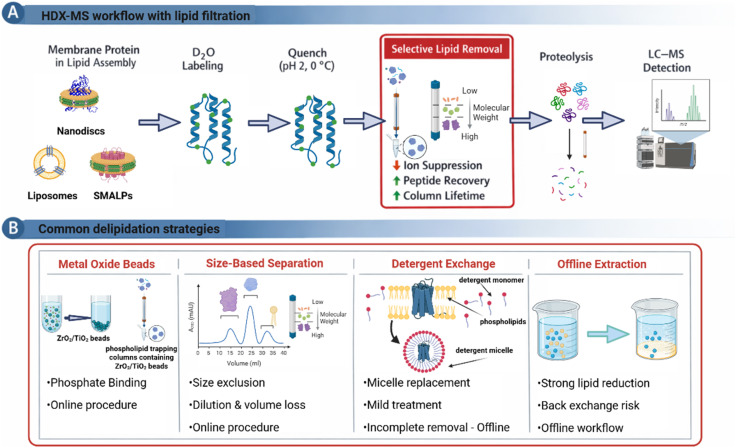
(A) HDX-MS workflow with online lipid removal method and (B) common delipidation strategies, both offline and online (phospholipid removal with metal oxide beads, size-based separation, detergent exchange, offline extraction), with strengths and limitations. Created in https://BioRender.com.

One of t11he main challenges when probing membrane protein dynamics with HDX-MS is chromatographic interference and ion suppression of lipid species in the mass spectrometer.^38^ Effective lipid removal is critical for protecting MS instrumentation, minimising signal suppression, and ensuring high-quality data (Fig. 2A). Different delipidation strategies have been proposed in the literature, such as offline liquid–liquid extraction^57^ and detergent exchange,^58^ and online lipid trapping with size-exclusion chromatography (SEC)^59^ and metal oxide (zirconium oxide (ZrO_2_)- and titanium oxide (TiO_2_)) beads,^20,60^ as summarised in Fig. 2B. In this perspective, we will comment only on advanced online delipidation strategies.

Automated PL filtration, where PLs bind strongly to metal oxides *via* phosphate groups, has emerged as a practical solution for minimising lipid-related interference in mass spectrometry workflows, enabling more robust analysis of membrane protein systems.^20,60^ Hammerschmid *et al.*^60^ presented an HDX-MS workflow in which a chromatographic PL trap column is incorporated directly into the instrument, enabling automated removal of lipids from deuterium-labelled protein–lipid assemblies prior to proteolytic digestion. The approach is validated using MSP-based nanodiscs, both in their empty form and when reconstituted with the ∼115 kDa transmembrane protein Acriflavine resistance protein B (AcrB). Efficient and reproducible PL capture was achieved with minimal deuterium back-exchange, low peptide carry-over, and negligible protein loss. In addition, a comparison of the performance of ZrO_2_- and TiO_2_-coated beads for lipid trapping was performed and showed how buffer composition and solution conditions can be tuned to improve lipid removal, benefiting both online and conventional offline delipidation strategies. Overall, this method substantially lowers practical barriers to HDX-MS analysis of membrane proteins, facilitating more detailed studies of their conformational dynamics in artificial bilayers and potentially even in native membrane environments.

Calvaresi *et al.*^59^ implemented a short SEC column into a standard, commercially available HDX-MS workflow, thereby introducing an additional separation step based on molecular size. The system was carefully optimised to limit nonspecific protein interactions with the SEC stationary phase and to maintain low levels of back-exchange. The SEC step is completed within minutes under quench conditions—low temperature and acidic pH—and its performance was systematically evaluated for separating both proteins and low-molecular-weight components, including lipids, across a broad mass range. Using a set of model proteins, it was shown that the SEC-HDX-MS approach effectively addresses several common analytical bottlenecks encountered in complex samples. These include the removal of lipids from protein–lipid assemblies, rapid desalting in heterogeneous matrices, and elimination of highly ionizable LC-MS interferents such as tris(2-carboxyethyl)phosphine (TCEP) from fully reduced, disulfide-rich proteins.

Recently, Guffick *et al.*^54^ developed a fully automated workflow, utilising a dual-mode delipidation step, for *in situ* HDX-MS analyses. The innovative platform uses ZrO_2_ and SEC-mediated delipidation in tandem and enables the identification of a high number of peptides from complex systems, integral membrane proteins, with minimal back exchange and no column degradation. Investigating the dynamics of an ATP-binding cassette (ABC) transporter, MsbA, *in situ* confirmed the physiological existence of an outward-facing (OF_open_) conformation and revealed previously unidentified dynamics within the cytoplasmic nucleotide binding domains.

Automated lipid filtration is a critical step for probing complex membrane protein systems by HDX-MS without the need for time-consuming manual operations, therefore online lipid removal is generally preferred over offline. The focus is on removing lipid molecules without disrupting protein integrity or deuterium labelling and achieving reduced analytical interference and back-exchange, improved data quality, and enhanced reproducibility. As such technologies continue to mature and become more widely adopted, they are likely to play a central role in lowering technical barriers to HDX-MS, allowing protein dynamics to be probed in membrane environments that more closely resemble physiological conditions. Removing lipids post-labelling helps minimise signal suppression, while the perturbation of the protein structure caused by the lipid is still captured.

### 3.2 Advanced instrumentation

**Fig. 3:**
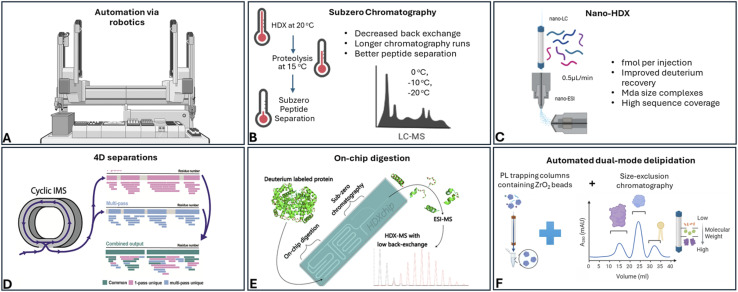
Advances in instrumentation for HDX-MS analyses. (A) Automation for enhanced reproducibility, (B) subzero chromatography, (C) nanoscale separations, (D) incorporation of ion mobility for 4D-separations, reproduced from ref. 67 with permission from American Chemical Society, copyright 2024, (E) on-chip digestion, reproduced from ref. 68 with permission from American Chemical Society, copyright 2025, and (F) fully automated dual-mode delipidation. Created in https://BioRender.com.

Recent trends in HDX-MS aim to strategically advance its capabilities for interrogating complex protein systems, such as membrane proteins, by enhancing separation capacity and sensitivity. There is high demand for automated processes *via* extended robotics (Fig. 3A). Manual HDX-MS experiments were the only way before automation emerged. The ability to enable high-throughput analyses and reduce variability has driven the development and incorporation of automation into HDX-MS. An early approach has been to focus on automation of the LC-MS analysis by using a −80 °C sample holder coupled to an autosampler for controlled thawing and injection of samples.^61^ Since then, more sophisticated cooling systems for automated thawing and LC-MS analysis of HDX-MS have been developed^62^ where HDX sample preparation is disconnected from the LC-MS analysis.

There has been development in automation approaches that cover both sample preparation and HDX-MS analysis steps. An early implementation of automated sample preparation used a sample handling robot to perform the deuterium labelling and quenching steps, followed by online digestion coupled to LC-MS.^17,63^ A limit of the early sample handling systems was the restriction in the time range it could sample due to the physical movements necessary for transferring and mixing solutions.

HDX-MS measurements are further complicated by pronounced spectral complexity, which stems from the inherent complexity of biological samples and the limited chromatographic resolving power imposed by the use of short LC gradients required to minimise deuterium back-exchange. To address these challenges, Venable *et al.*^19^ introduced subzero temperature chromatography coupled to HDX-MS (Fig. 3B). By operating prolonged reversed-phase separations at temperatures of −10 °C, −20 °C, and −30 °C (using methanol/ethylene glycol, formamide, and dimethylformamide-modified mobile phases), the method enhances peptide separation without increased back-exchange, improving therefore sequence coverage, enabling more accurate and detailed analysis of protein structural changes. Especially for membrane proteins—where sequence coverage is often limited and subtle lipid-induced conformational changes must be resolved—such enhanced deuterium retention is particularly critical. Later, Wales *et al.*^64^ constructed a novel system that allows controlled temperature through digestion, trapping and separation. To avoid solvent freezing during subzero separations, 35% methanol was added to the mobile phase immediately after online digestion. Lowering the separation temperature from 0 °C to −20 °C did not significantly compromise chromatographic efficiency but led to improved deuterium retention. A 10 min gradient at 0 °C yielded an average deuterium recovery of 77% for phosphorylase b tryptic peptides, whereas analysis of the same sample using a 1 h gradient at −20 °C increased average deuterium recovery to 82%. Recently, Fang *et al.*^65^ developed a long gradient subzero-chromatography (−10 °C) for HDX-MS analysis of conformational dynamics of complex peptide mixtures. Roughly at 90 min, a threefold increase compared with a 15 min gradient run under the same temperature was achieved. Peptides eluting later in the gradient during the 90 min run preserved high levels of deuterium incorporation even at the tail end of the separation. Subzero temperature HDX-MS can significantly transform our ability to study membrane proteins and complex cellular systems by dramatically reducing back-exchange and allowing longer gradients without compromising deuterium labelling, which in turn allows higher sequence coverage and improved sensitivity for low-abundance or challenging peptides.^66^

Recent advancements in LC systems have enabled the use of the nanoLC coupled to HDX. A commercial HDX platform has been modified for nanoflow separations (nanoHDX, Fig. 3C), providing a significant increase in sensitivity that is 100 times greater than that of conventional microflow HDX setups.^21^ Additionally, this arrangement reduces back-exchange and more successfully preserves dynamic information. Interestingly, femtomole amounts of material can be used to examine huge assemblies on the megadalton scale, resulting in broad sequence coverage. nanoHDX was designed for high-throughput analysis of previously inaccessible protein systems, owing to its drastic sample requirements reduction and rapid re-equilibration, which allows processing up to 80 samples per day. The method is simple to apply, enabling the whole structural biology community to use high-performance HDX-MS.

There has been advancement and novel instrumentation for performing fast chromatographic sample clean-up and separation. In this direction, incorporating ion mobility (IM) into HDX-MS workflows improves data quality by adding an orthogonal, gas-phase separation of peptide ions. Griffiths *et al.*^67^ reported for the first time an HDX-MS strategy that incorporates both single-pass and multipass cyclic IM separations (Fig. 3D), resulting in a 31–222% increase in overall peptide output compared with earlier platforms and up to a 42% rise in peptide identifications for individual targets, thereby enhancing sequence coverage and redundancy for challenging membrane protein samples.

In terms of sample pretreatment, innovative materials featuring microfluidic chips have been newly developed (Fig. 3E), capable of rapid on-chip protein labelling with deuterium and subsequent sample quenching.^68,69^ Thiol-ene microfluidic chips have been used to perform HDX in 0.14–1.1 s with high reproducibility, significantly advancing the capability to probe transient conformational dynamics in solution. Recently, a subzero cooled microchip (HDXchip) was developed that integrates proteolysis, desalting, and chromatography for HDX-MS while greatly reducing deuterium back exchange compared to conventional systems.^68^ The authors mentioned high sensitivity and sequence coverage for both global and local analyses of model proteins, such as hemoglobin, and highlighted the potential of low-temperature microfluidics to improve HDX-MS performance.

A recent preprint, currently under revision by Guffick *et al.*^54^ reported a fully automated workflow utilising a dual mode delipidation workflow (DMD), demonstrating that automated HDX-MS can routinely probe membrane protein dynamics directly within native membrane vesicles (Fig. 3F). They reconfigured a dual-head LEAP HDX robot with a filtration system to perform all sample handling and mixing, ZrO_2_- and SEC-mediated delipidation for the first time, without affecting analytical throughput. To test the DMD, the authors employed 1-palmitoyl-2-oleoyl-*sn*-glycero-3-phosphocholine (POPC) as a model system to test for removal efficiency. ZrO_2_ delipidation alone removed up to 100 pmol of POPC with 73% efficiency, SEC-mediated delipidation successfully removed 98% of 2500 pmol POPC, while the DMD workflow removed 99% of 3500 pmol POPC. Although back exchange was higher (47 ± 3%) with the DMD delipidation workflow than with workflows lacking a delipidation step, the increase was less than 5% and remained within acceptable limits.^70^ The broader significance is a shift from studying membrane proteins in simplified membrane mimetics toward characterising them in physiologically relevant lipid environments.

Overall, recent trends in HDX-MS instrumentation, as illustrated in Fig. 3, enable the shaping of the conformational dynamics and function of difficult-to-probe complex membrane-associated protein systems. Improvements in separation technologies include 4D separations with IM and the development of subzero and nanoflow configurations to address long-standing challenges such as spectral overlap, deuterium back-exchange, and decreased sensitivity. At the same time, the development of highly automated platforms, microfluidic devices, and chip-based workflows has significantly simplified sample preparation and enabled precise control over labelling, quenching, and digestion on increasingly short timescales. Although each of these innovations represents a significant technological advance, their integration into a unified, fully consolidated platform remains an unresolved challenge. Collectively, however, all these technological advances are moving HDX-MS beyond a niche method and into a more robust, high-throughput approach for probing large protein assemblies, working with limited sample amounts, and operating in native-like environments, allowing for the first time to address biological questions that were previously out of reach.

### 3.3 Native environment

Understanding how membrane proteins interact with lipids remains a major challenge, as in their native setting, these proteins are embedded within complex and heterogeneous lipid environments that strongly influence their structure, dynamics, and function. The ultimate goal is to probe protein–lipid interactions either *in situ* or *in vivo*. In this direction, Rey *et al.*^71^ investigated the conformational dynamics of bovine adenine nucleotide carrier isoform 1 *in situ* in mitochondria. The *in situ* HDX-MS measurements revealed differential deuterium uptake patterns that reflect the dynamic behaviour of the carrier in its functional lipid environment, providing insights into how conformational changes relevant to transport are modulated by the surrounding membrane context. Mehmood *et al.*^55^ studied how a multidrug ABC transporter adopts different conformational states during its functional cycle. By comparing the transporter in a nucleotide-free resting state with an adenosine triphosphate (ATP) bound state in which the nucleotide-binding domains associate, the authors probed how structural dynamics change between these two states. Global HDX-MS measurements, together with limited proteolysis, revealed that the transporter adopts two clearly distinct conformations both in membranes and in detergent. One state is compact and highly protected, showing low deuterium uptake and strong resistance to protease digestion, while the other is markedly more flexible and accessible, displaying increased exchange and protease sensitivity. These observations provide direct experimental support for a transport mechanism in which multidrug ABC transporters alternate between an inward-facing open resting conformation and a transient, outward-facing closed state stabilised by ATP binding. In another study,^72^ a newly developed protocol was applied for probing the conformational outer membrane protein F (OmpF) from outer-membrane vesicles naturally released by *Escherichia coli* (*E. coli*). Peptide coverage was calculated 85.9%, with the highest difference in deuterium incorporation in the three experiments not exceeding 2%, demonstrating method reproducibility. Studies with and without lipid removal showed that lipid cleanup had a negligible impact on the HDX labelling pattern, confirming that the method preserved meaningful structural information while removing problematic lipids.

While studying membrane proteins in native membranes is a significant advancement for HDX-MS, it could not have all the characteristics found in complete cells, such as the existence of possible downstream partners. Lin *et al.*^14^ developed a novel protocol conducting HDX-MS on the vitamin B12 transporter in live *E. coli* cells. Live *E. coli* cells were transferred to deuterated growth media to label the protein. Following labelling, the process was stopped, and cryogenic pulverisation was used to lyse the cells. Under quenching conditions, ultracentrifugation was used to separate the membranes, and HDX-MS was applied, reaching 93% sequence coverage with the disadvantage of greater back exchange due to the long post-quench processes. For one peptide, it was noted that the maximum deuterium incorporation decreased from 78% (*in vitro*) to 28% (*in vivo*). The authors also acquired HDX data on several other endogenous *E. coli* proteins.

Together, these results established HDX-MS as a sensitive tool for protein structure and dynamics, where differences in deuterium uptake directly reflect variations in hydrogen bonding, solvent accessibility, and conformational flexibility. The high sequence coverage and excellent reproducibility achieved across independent experiments showed that *in situ*/*in vivo* HDX can reliably capture both rapidly exchanging and more protected regions of membrane proteins. While the proof-of-principle study by Lin *et al.*^14^ demonstrated that *in vivo* HDX-MS is feasible, several technical hurdles still prevent its routine implementation. The principal limitation is the extensive post-quench sample processing required before MS analysis, which must all be performed under conditions that minimise deuterium back-exchange. These procedures are considerably longer and more complex than those used for purified proteins, increasing opportunities for deuterium loss and experimental variability. Sample purification, which had occurred during the period between cell harvesting and initiation of HDX, is now conducted post-HDX labelling and under quench conditions. This necessitates optimisation of the protocol to strike a compromise between sample quantity, purity, and back exchange. In addition, efficient cell disruption presents a major challenge. Cell lysis must be sufficiently rapid to preserve the deuterium labelling pattern while ensuring complete recovery of membrane proteins without altering their native conformational state.

Membrane proteins are typically present at low abundance, which in the cell makes their analysis even harder since they coexist with other proteins. This complicates peptide identification and may subsequently reduce sequence coverage due to overlapping peptides and low signal-to-noise. Consequently, enrichment strategies and highly efficient online clean-up methods are required before HDX-MS analysis. *In vivo* HDX-MS should be performed without overexpression to preserve the relative protein concentrations, which may be possible with future optimisation of the back exchange levels and increased sensitivity of instrumentation. Future progress will therefore depend on advances in rapid, automated post-quench processing, such as a purification tag that functions at low pH, online delipidation, and high-sensitivity instrumentation. Together with improved computational workflows for analysing increasingly complex datasets, these developments are expected to transform *in vivo* HDX-MS from a proof-of-concept methodology into a routine tool for studying protein dynamics directly in living cells.

## 4 Integration with other MS techniques

### 4.1 Lipidomics

Lipidomics is an emerging area of research for understanding lipid metabolism and the active role lipids play in disease. It is applied to identify endogenous lipids retained by membrane proteins.^73^ Lately, the development of innovative workflows for untargeted and targeted lipidomics, enabling high coverage of polar metabolites and non-polar lipids within one analytical run, has become of increasing interest. Recent trends include the introduction of microflow columns (1.0–2.1 mm internal diameter)^74^ and IM-MS^75^ that, together, enable high-throughput analysis, allowing for the identification of hundreds of lipids in ∼10 min runs with minimal sample and solvent consumption. Single-cell lipidomics has also emerged as a powerful approach to investigate cellular lipid metabolism, providing insights into dynamic lipid profiles, cell-specific signalling pathways, and the molecular changes associated with disease progression.^76–78^

HDX-MS in combination with lipidomics have been applied to study membrane protein conformational dynamics within native lipid bilayers.^57,79^ Reading *et al.*^79^ coupled HDX-MS with detailed lipid profiling to investigate changes in conformational dynamics of the rhomboid protease GlpG in correlation to three different lipid compositions captured in SMALPs. This combination enabled mapping of protein regions sensitive to different lipid environments and linking these dynamic signatures to the identities of co-extracted lipids, thereby providing a more holistic view of how membrane lipids modulate protein structure and function than either technique could achieve alone. Liu *et al.*,^57^ reported the discovery of the natural small molecule cucurbitacin B (CuB) as a selective inhibitor of the mitochondrial enzyme phosphoenolpyruvate carboxykinase 2 (PCK2), which is over-expressed in hepatocellular carcinoma and linked to poor prognosis in patients. Disrupted mitochondrial membrane integrity and altered lipid profiles were observed by targeted lipidomics studies, showing a clear link between protein regulation and the surrounding lipid environment. This work indicates how advanced lipidomics can bridge changes in protein function with measurable shifts in lipid composition—complementing approaches like HDX-MS that probe protein dynamics. HDX-MS revealed that CuB allosterically stabilises the PCK2 lid domain, altering its conformational dynamics in ways that correlate with lipidomic changes and disruptions in mitochondrial membrane integrity, highlighting the power of HDX-MS to capture allosteric modulation of dynamic protein elements in a native-like environment.

HDX-MS provides valuable insights into how protein regions respond dynamically to changes in their environment, but on its own, it cannot define which lipid species are responsible for these effects. Lipidomics is a valuable tool that provides this missing chemical context by identifying and quantifying lipids that co-purify with membrane proteins or are retained in native-like assemblies. Together, these methods enable the interpretation of protein dynamics changes found by HDX-MS in terms of particular lipid interactions as opposed to nonspecific membrane effects. In a manner that is challenging to accomplish with either approach alone, this combined approach provides a more realistic and mechanistic understanding of membrane protein regulation by directly connecting lipid identity to structural and functional results.

### 4.2 Native MS

Native MS exploits the gentle nature of nano electrospray ionisation (nanoESI) to transfer proteins and their noncovalent complexes from volatile buffered aqueous solutions into the gas phase, thereby preserving and enabling direct analysis of tertiary and quaternary structure, heterogeneity, and oligomerisation.^80^ Although native MS was initially primarily used to study soluble protein assemblies, membrane protein complexes have also entered the realm of native MS through electrospraying from membrane mimetics, or even native lipid membranes.^81,82^ Native MS is effectively applied for gaining profound structural insights, to distinguish conformational families *via* charge state distributions (folded *versus* unfolded),^83^ to detect the stoichiometry of complexes,^84^ changes in the distribution of oligomeric states as a function of lipid/ligand concentration,^85^ and measure affinities of noncovalent protein–lipid interactions.^86^

The data from native MS and HDX-MS can be brought together to build detailed structural models of membrane protein complexes, giving a clearer picture of how they function in their native environment.^87^ Native MS provides intact quaternary structure information for protein assemblies, while HDX-MS reports on conformational dynamics and solvent accessibility, making the two techniques complementary for comprehensive analysis of protein states and complexes. Native MS is capable of distinguishing annular from non-annular lipids based on lipid residence time and resistance to detergent competition,^88^ providing information inaccessible through HDX-MS. Alone by measuring detergent-mediated lipid displacement, native MS can distinguish rapidly exchanging annular lipids from specific, tightly bound non-annular lipids that occupy functionally important binding sites. HDX-MS is most sensitive to long-lived, site-specific lipid interactions and is therefore particularly well suited for investigating non-annular lipid binding. This complementarity is in its early stage due to the lack of commercial mass spectrometers that can span multiple applications, including both-omics and native experiments, together with the lack of experienced users for both applications.

The power of combining these two MS techniques has been proven using soluble proteins^89–94^ with some recent publications on membrane protein–lipid interactions. An early study paving the way towards combinatory native MS and HDX-MS studies on membrane protein–lipid interactions is by Demmers *et al.*^95^ where they used native MS as an analytical platform to measure HDX kinetics of transmembrane peptides embedded in lipid bilayers. By combining deuterium labelling with native MS readout, the authors track backbone amide exchange rates to probe solvent accessibility and structural dynamics of membrane-associated peptides. The study demonstrates that native MS can reliably resolve deuterium uptake differences for peptides in different lipid environments, providing insights into peptide conformation, membrane insertion, and local dynamics.

Derek's group has taken advantage of the strengths of each technique to study protein–lipid interactions. They examined how interactions with cardiolipin and phosphatidylcholine PLs remodel the conformational ensemble of cytochrome c.^96^ Native MS and IM-MS revealed lipid-dependent binding stoichiometries and shifts in charge-state distributions, indicating distinct populations of cytochrome c stabilised by each PL, while millisecond time-resolved HDX-MS (TRESI-HDX-MS) mapped lipid-specific changes in backbone dynamics. Together, the native MS and HDX-MS data demonstrated that cytochrome c was observed to adopt a more compact configuration when bound to cardiolipin, in contrast to binding phosphatidylcholine, providing a mechanistic link between lipid binding, protein dynamics, and functional switching in mitochondrial membranes. Later, they used native MS and TRESI-HDX-MS to monitor how α-synuclein (αSN), an intrinsically disordered protein, changes shape in real time when interacting with PL nanodiscs.^97^ Native MS spectra of αSN and MSP1D1 ΔH5 were obtained in order to confirm complexation, the protein identity, check for any impurities or degradation products, and view folded and unfolded parts of the protein. TRESI-HDX-MS data showed that upon binding to lipid nanodiscs, αSN exhibits site-specific decreases in deuterium uptake in regions known to mediate membrane contact, reflecting rapid stabilisation and folding of those segments. Conversely, other regions remain highly dynamic, highlighting the heterogeneous conformational ensemble of αSN on the membrane surface. The combined MS approaches demonstrate that lipid binding induces conformational ordering in specific αSN regions, while preserving flexibility in other parts – insights relevant to its physiological membrane binding and pathological aggregation.

## 5 Future directions

Recent developments in HDX-MS have made clear that it may play a vital, and rather unique, role in structural biology, providing detailed information about the effects on protein structure and function due to changes in the environment and lipid binding. From a highly specialised tool for studying simple proteins, there have been analytical advances over the past three decades that allow HDX-MS to become a commercialised tool that is used to analyse complex membrane proteins. HDX-MS has revealed critical insight into protein dynamics, folding pathways, and ligand/lipid interactions, continuously expanding the utility of HDX-MS. The increased accessibility of high-resolution mass spectrometers, user-friendly software, and commercial automated options for HDX-MS have lowered the barrier to entry for new users to apply HDX-MS to more diverse systems, making the study of large molecular complexes feasible.

The ultimate goal of many structural techniques is to be able to study intact complexes without disturbing the native lipid environment.^98^ Lipid vesicles and membrane mimetics are our best shot currently, but challenges remain—particularly due to lipid-induced ion suppression and chromatographic interference. In this context, online delipidation strategies, full automation, and orthogonal IM separations pave the way for tackling the conformational dynamics of challenging membrane proteins *in vivo*.

Future techniques should attempt to move away from detergents and other mimetics, given that membrane protein dynamics are so closely linked to the native membrane environment.^99^ As research turns to eukaryotic membrane proteins with complex proteoforms and a high degree of post-translational modifications, such as phosphorylation and glycosylation, significant analytical developments will be needed to reduce heterogeneity and improve the instrumentation and experimental approach to handle complex samples. Furthermore, eukaryotic membrane proteins may be less stable, so until *in situ* or *in vivo* studies are possible, new membrane mimetic strategies may be required to preserve the activity of these delicate proteins for HDX-MS. A key factor in these advances is instrumental developments in activation, IM, and mass analysers, especially in novel combinations of these methods. Novel instrumentation, improved automation that allows online lipid filtration and desalting, such as ZrO_2_, microchip and online SEC, and increased peak capacity with the use of subzero LC, will undoubtedly enable novel experiments and tackle more challenging membrane protein targets under complex physiological conditions. Furthermore, the development of nano flow HDX-MS dramatically decreases sample consumption, while peptide separation efficiency and, hence, protein sequence coverage and redundancy are greatly improved, a crucial aspect for membrane proteins. Subzero chromatography may make it possible to investigate the dynamics of systems that have previously been challenging to investigate using conventional HDX-MS, while minimising back-exchange and maintaining high sequence coverage. Although the integration of subzero workflows poses technical challenges, such as preserving protein stability during sample introduction, this technology is positioned as a crucial next step for structural proteomics due to its potential to significantly improve data quality.

The data analysis stage has historically been a bottleneck for HDX-MS studies. The combination of peak integration, isotopic peak-picking, and deuterium incorporation analysis for several time points, replicates, and multiple conditions is time-consuming and requires high computer power even for a single peptide. When considering dozens to hundreds of peptides, the magnitude of data analysis can be daunting. For many years, the inability to cope with larger data sets was one of the main factors limiting the size of proteins amenable to HDX-MS. A variety of software options have emerged and have been continually refined to accommodate the need for the analysis of larger HDX-MS data sets.^100^ Modern algorithms generally offer sophisticated peak picking, isotopic distribution fitting, and quality assessment of data, limiting the required manual analysis to predominantly curation and validation. Today, there are several established HDX-MS software options, both academic and commercial. These software options have some subtle differences regarding their included features or their approach for picking species of interest to calculate deuterium incorporation from the isotopic profiles. Several of these programs include tools for the extraction and integration of the data from raw MS acquisition files, while others rely on external programs for data extraction from the acquisition files and are therefore used primarily for deuterium calculation or visualisation of the results. Some software only support specific data file formats, limiting their use to certain instrument types and vendors.^101–103^

MS/MS fragmentation most commonly follows a data-dependent acquisition (DDA) mode, wherein precursor ions are selected for fragmentation based on their abundance and charge. While DDA is the most used mode, reproducibility of precursor selection and long instrument cycle times limit the applicability of DDA for HDX. To address DDA shortcomings, the data-independent acquisition (DIA) mode was introduced, wherein all ions within a small isolation window are fragmented despite their abundance or charge by relying on fast fragmentation to generate sequence data across the entire mass range, and at a rate sufficient to support the chromatographic timescale of HDX-MS.^104^ This produces an unbiased but complex MS/MS spectra, for deconvolution to match the fragment ions to the precursor. Deuteration data can then be measured with a recently developed software from the precursor ions and from the fragments, provided they have suitable ion statistics.^101^ While DIA offers advantages when compared to DDA, an inherent limitation with bottom-up HDX-MS studies has been peptide-level spatial resolution, where the measured deuterium level reflects the sum total of all exchange at each backbone amide in a peptide (average length is 12–13 residues). While the analysis of overlapping peptides can provide increased spatial resolution,^105^ many studies have also explored electron-based fragmentation approaches, electron capture dissociation (ECD),^8^ or electron transfer dissociation (ETD),^106,107^ to measure deuterium levels in fragment ions and thereby localise deuterium to much smaller sequences, perhaps even single residues. With the increasing accessibility to electron-based fragmentation approaches and continued software advances, single-residue resolution HDX-MS may become routine. The increased availability of instrumentation capable of electron- or photon-based dissociation, together with continued advances in machine learning such as PFNet model.^108^ and likely additional novel approaches for peptide fragmentation in the future, is expected to propel the use of site-specific HDX-MS studies in the coming years. PFNet enables rapid determination of residue-level free energies of opening (Δ*G*_op_) from conventional peptide-level HDX-MS datasets.

Currently, molecular dynamics (MD) simulations can reach up to milliseconds of protein trajectory, while HDX can be performed over hours or days. It is extremely challenging to simulate the exchange on a comparable time scale. With an improved understanding of the factors governing HDX and the advancement of computing technologies, it is possible to achieve closer correlations between the dynamic information obtained by MD simulation and HDX.^109^ This combination allows for predicting lipid-sensitive regulatory regions, identifying potential allosteric/druggable conformations, and integrating structural models (*e.g.*, AlphaFold). A major challenge is to translate the HDX data into structural information so as to be able to formulate conclusive mechanistic insights. With HDXer, a distribution of conformations in a pre-existing ensemble is reweighted *post hoc* so that calculated ensemble-averaged deuteration levels match a given set of target data.^110^ HDXer uses maximum-entropy reweighting of simulated structural ensembles to quantitatively interpret HDX-MS measurements, enabling mechanistic insights into protein conformational populations.^111^ Recently, an artificial intelligence (AI)-HDX^112^ workflow was enabled for the first time to uncover protein structural dynamics by combining deep learning, experimental HDX, sequence alignment, and protein structure prediction. AI has also been used for automatic HDX-MS data correction for back and forward exchange using models generated by deep neural networks.^113^

**Fig. 4:**
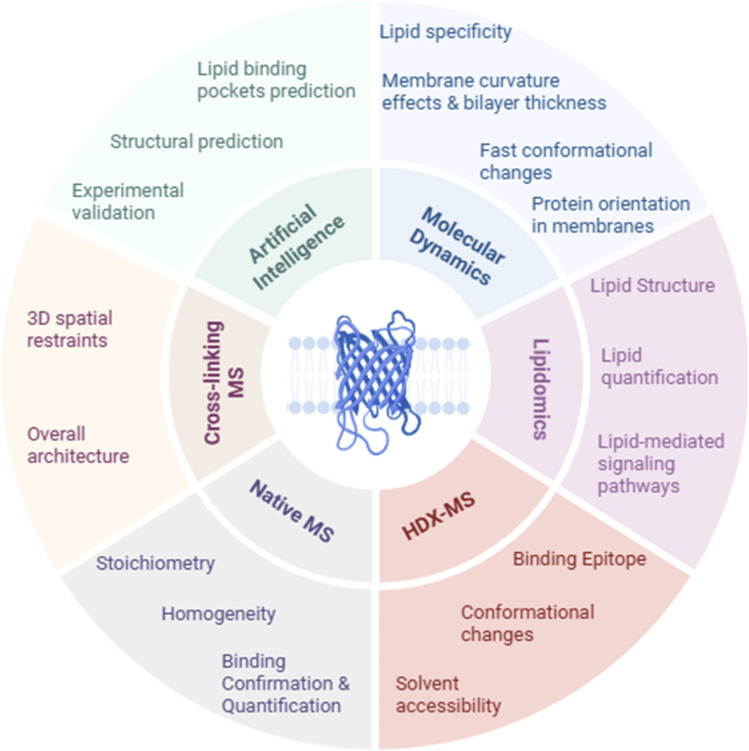
Integrating mass spectrometry, molecular dynamics simulations, and AI-based structure prediction to characterise membrane protein conformational landscapes and lipid interactions. Created with https://BioRender.com.

The future of protein dynamics research is promising, particularly when HDX-MS is integrated with complementary approaches such as other MS techniques, AI, and MD simulations, maximising structural and functional insights, as summarised in Fig. 4. For instance, crosslinking-MS has been extensively applied to identify protein–lipid interactions, particularly through photoaffinity labelling strategies.^114,115^ Several investigations have also employed *in situ* crosslinking-MS to characterise protein interaction networks.^116–118^ However, only a limited number of studies have combined crosslinking-MS with HDX-MS to gain complementary structural insights, and none of these has focused specifically on protein–lipid interactions.^100,119^ Therefore, we expect that future methodological advances will further promote the integration of these approaches. Finally, we anticipate an increasing use of other structural techniques such as cryo-EM, X-ray crystallography, and NMR. Continued improvements in separation technologies and MS sensitivity will undoubtedly propel HDX-MS to address even more sophisticated and exciting biological questions to the point where studying protein dynamics and interactions within living cells might be possible by HDX-MS.^38^ With an established methodology of cellular lipidomics^120,121^ and something that has already been demonstrated for native MS by Chorev *et al.*^37^ who have released proteins directly from native membranes into the gas phase of the mass spectrometer, a method called SOLVE (sonicated lipid vesicles). New interactions were uncovered that HDX-MS can shed light on the dynamics.

A decade ago, the primary bottleneck in HDX-MS was instrumentation. Limited automation reduced experimental reproducibility, contributed to significant deuterium back-exchange and limited throughput. Instrumentation is still improving, but it is no longer the primary limitation. Today, sample preparation of membrane proteins remains the largest experimental bottleneck. Preserving native lipid interactions while simultaneously removing enough lipid to obtain high-quality MS data remains fundamentally difficult. No membrane mimetic is universally applicable, and each introduces compromises between biological relevance and analysis. The advancements in delipidation and instrumentation are reducing this gap, but robust, standardised sample preparation for native membrane systems remains elusive.

Among the aforementioned, emerging future directions, the increasing availability of electron-based fragmentation methods, combined with advances in machine-learning, is expected to make single-residue resolution HDX-MS increasingly accessible and routine. As machine-learning approaches become more deeply integrated across the life sciences, they are likely to play an increasingly important role in HDX-MS experimental design and data interpretation. Under critical evaluation by the researcher, these tools could accelerate method optimisation, guide the selection of experimental parameters such as quench conditions and delipidation strategies, and ultimately improve the efficiency, robustness, and reproducibility of HDX-MS workflows. HDX-MS will further grow as applications, methodologies, and instrumentation expand, fostering a greater depth of information that can be gained for membrane protein analysis.

## 6 Conclusions

HDX-MS has emerged as a powerful and versatile approach for probing the dynamic interplay between membrane proteins and their surrounding lipid environments. Over the past decades, significant experimental and instrumental developments have advanced HDX-MS into an accessible platform capable of analysing complex membrane protein assemblies under increasingly native-like conditions. Several questions that were nearly impossible only a few years ago are now beginning to be addressed. HDX-MS can now reveal how a lipid changes protein dynamics. Improved instrumentation now allows better resolution and higher sequence coverage, enabling HDX-MS to probe dynamic states with higher detail. Studying protein dynamics directly inside living cells still remains an ambitious goal, but proof-of-principle *in vivo* HDX-MS studies have already demonstrated its feasibility. Looking ahead, the scientific community would definitely benefit from the transition from a standalone structural method to an integrative structural biology platform to provide a more comprehensive understanding of membrane protein function in native environments.

The integration of HDX-MS with native MS, lipidomics, and computational approaches promises a more holistic view of membrane protein function. Such multidimensional strategies will be essential for understanding the complex, dynamic nature of biological membranes and providing critical insights into protein–lipid interactions, allosteric regulation, and conformational heterogeneity. This has a direct impact on drug discovery, understanding disease-related dysfunctions, and deciphering fundamental mechanisms of membrane protein regulation and signalling pathways.^122^ Despite these advances, several challenges remain. Membrane protein heterogeneity, instability in non-native environments, limited spatial resolution, and the complexity of data analysis continue to restrict broader adoption and high throughput. Addressing these limitations will require further developments in automation, separation technologies, fragmentation methods, and integrated software platforms capable of handling increasingly complex multidimensional datasets. As sensitivity, resolution, and throughput continue to improve, HDX-MS is expected to move beyond reconstituted systems toward the study of membrane proteins directly within native membranes, organelles, or even living cells.

## Author contributions

All authors contributed to the first draft and approved the final version of the manuscript.

## Conflicts of interest

The authors declare that they have no conflicts of interest.

## Data Availability

No primary research results, software or code have been included, and no new data were generated or analysed as part of this review.
